# Phosphodiesterase-2 Inhibitor Bay 60-7550 Ameliorates Aβ-Induced Cognitive and Memory Impairment via Regulation of the HPA Axis

**DOI:** 10.3389/fncel.2019.00432

**Published:** 2019-10-02

**Authors:** Lina Ruan, Kai Du, Mengjia Tao, Chunyan Shan, Ruixuan Ye, Yali Tang, Hanbo Pan, Jinpeng Lv, Meixi Zhang, Jianchun Pan

**Affiliations:** ^1^Brain Institute, School of Pharmacy, Wenzhou Medical University, Wenzhou, China; ^2^Ningbo Key Laboratory of Behavioral Neuroscience, Zhejiang Provincial Key Laboratory of Pathophysiology, School of Medicine, Ningbo University, Ningbo, China; ^3^College of Pharmaceutical Engineering and Life Sciences, Changzhou University, Changzhou, China; ^4^Pingyang County Hospital of Traditional Chinese Medicine, Pingyang County, China

**Keywords:** beta amyloid 1–42, phosphodiesterase 2, Bay 60-7550, hypothalamus–pituitary–adrenal axis, pCREB, BDNF

## Abstract

The dysfunction of the hypothalamus–pituitary–adrenal (HPA) axis is often seen in Alzheimer’s disease (AD) patients with cognitive deficits. Selective inhibition of phosphodiesterase (PDE) 4 and 5 has already proven to be effective in reducing beta-amyloid 1–42 (Aβ1–42)-mediated pathology by regulating corticotropin-releasing factor (CRF) and glucocorticoid receptor (GR) expression, suggesting that PDE-dependent signaling is involved in Aβ1–42-induced HPA axis dysfunction. However, nausea and vomiting are the side effects of some PDE4 inhibitors, which turn our attention to other PDEs. PDE2 are highly expressed in the hippocampus and cortex, which associate with learning and memory, but not in the area postrema that would cause vomiting. The present study suggested that microinjection of Aβ1–42 to the intracerebroventricle induced learning and memory impairments and dysregulation of the HPA axis by increased expression of CRF and GR. However, the PDE2 inhibitor Bay 60-7550 significantly ameliorated the learning and memory impairment in the Morris water maze (MWM) and step-down passive avoidance tests. The Aβ1–42-induced increased CRF and GR levels were also reversed by the treatment with Bay 60-7550. These Bay 60-7550’s effects were prevented by pretreatment with the PKG inhibitor KT5823. Moreover, the Bay 60-7550-induced downstream phosphorylation of cyclic AMP response element binding (pCREB) and brain-derived neurotrophic factor (BDNF) expression was also prevented (or partially prevented) by KT5823 or the PKA inhibitor H89. These results may lead to the discovery of novel strategies for the treatment of age-related cognitive disorders, such as AD, which affects approximately 44 million people worldwide.

## Introduction

Alzheimer’s disease (AD) is a progressive neurodegenerative disease characterized by the accumulation of beta-amyloid peptides (Aβ), neurofibrillary tangles in the brain, widespread cortical neuronal loss, and progressive memory impairment. The major factors involved in AD-related cognitive impairment are unknown, but increasing evidence suggests that chronic stress-induced abnormal aging is one of the events that contribute to cognitive decline (Blennow et al., 2006; Querfurth and Laferla, 2010). These findings are consistent with the “glucocorticoid cascade hypothesis,” which suggests that AD patients with cognitive deficits are associated with hypothalamus–pituitary–adrenal (HPA) axis dysfunction, as evidenced by increased glucocorticoid (GC) levels and abnormal glucocorticoid receptor (GR) expression (De Kloet et al., 2005; Xu et al., 2009), leading to emotional (depression or anxiety) and cognitive disorders at the late stage of AD. In an acute animal model of AD, microinjection of an oligomeric solution of a Aβ fragment (oAβ) into cerebroventricle (i.c.v.) of animals induces multiple signs of neurodegeneration, which suggests a clear parallel with numerous relevant symptoms of AD patients (Zussy et al., 2011, 2013; Wang et al., 2012; Pineau et al., 2016). Indeed, this Aβ fragment originates from the proteolysis of parent amyloid proteins (Kubo et al., 2002; Gruden et al., 2007), which initiates a strong and long-lasting activation of the HPA axis, leading to cognitive and learning and memory impairment. Our recent study suggested that microinjection of Aβ1–42 induced memory impairment peaked at 2 months, which is associated with HPA axis dysfunction such as enhancement in serum corticosterone (CORT) level, corticotropin-releasing factor (CRF) expression, and GR expression in the brain. However, phosphodiesterase 4 (PDE4) inhibitor rolipram reversed this memory deficit through regulation of CRF and GR expression, suggesting that PDE-dependent signaling is involved in Aβ1–42-induced HPA axis dysfunction (Xu et al., 2013, 2015).

Cyclic AMP and cGMP are the 2 s messengers contributing to intracellular signaling transduction and gene transcription in learning and memory (Suvarna and O’Donnell, 2002). Compared to PDE4 inhibitors, PDE2 is a dual-substrate PDE enzyme that hydrolyzes both cAMP and cGMP. It is highly expressed in the limbic system, hippocampus, and cortex, and the adrenal cortex (Van Staveren et al., 2003) regions are associated with cognitive functions and HPA axis regulation, and it is not in the area postrema that is related to vomiting. Our previous study suggested that PDE2 potent inhibitor Bay 60-7550 reversed chronic stress-induced impaired cognition and depression-like behavior (Ding et al., 2014; Xu et al., 2015). However, it is not clear whether PDE2 inhibitors ameliorate Aβ relevant memory impairment through the regulation of the HPA axis system.

The present study investigated the memory-enhancing effects of the PDE2 inhibitor Bay 60-7550 on Aβ1–42-induced loss of learning and memory. The HPA-axis-related parameters, such as GR and CRF receptor expression, the phosphorylation of cyclic AMP response element binding (pCREB), and brain-derived neurotrophic factor (BDNF) levels were also investigated to elucidate how PDE2 inhibition could ameliorate memory and cognition through the regulation of HPA axis function.

## Materials and Methods

### Animals

ICR mice (male, 8 weeks, 22–25 g) were used (Chinese Academy of Sciences, China) for the experiments. Mice were allowed at least 5 days for habituation before the researches. According to the “NIH Guide for the Care and Use of Laboratory Animals,” all procedures were accepted by the animal care and use committee of Wenzhou Medical University.

### Surgery

The brain surgery was executed aseptically under ketamine (100 mg/kg, i.p.) and xylazine (10 mg/kg, i.p.) anesthesia. Mice were put in a stereotactic device with a flattened skull, and the head was kept horizontally. Bilateral guided intubation (30 gauge) was planted into the intracerebroventricle (anterior–posterior -1.7 mm from bregma, mediolateral ±0.8 mm from the midline, dorsoventral -2.0 mm from dura) for microinfusion. The total 2 μl of Aβ1–42 (0.4 μg/μl, 1 μl/each) was infused at a rate of 0.25 μl/min at 3 days after surgical treatment.

### Chemicals and Drug Administration

Aβ1–42 was obtained from rPeptide. Bay 60-7550 (CAS: 439083-90-6) was obtained from Sigma-Aldrich. The mice were treated with different doses of Bay 60-7550 (0.5, 1.0, 3.0 mg/kg/day, i.p.) or vehicle for 14 days after microinjection of Aβ1–42. PKG inhibitor KT5823 (Cayman Chemical, United States) and PKA inhibitor H89 (Sigma-Aldrich, United States) were microinjected bilaterally into the cerebroventricular, 30 min prior to treatment with Bay 60-7550. Antibodies against CRF, GR, p-CREB, CREB, and BDNF were obtained from Abcam (Cambridge, MA, United States). All secondary antibodies were obtained from Beyotime Biotechnology (Haimen, China).

### Behavioral Tests – Morris Water Maze Test

The Morris water maze test (MWM) was carried out as described previously (Xu et al., 2015). Briefly, a round pool was filled with opaque water with a ringed platform (155 mm high and 85 mm diameter) submerged 20 mm under the water in the first quadrant. Prior to the probe examination, six learning blocks were gathered and examined at an interval of 20 min. Each block comprised three tests, during which the mice were put in the instrument from the second, third, or fourth quadrant. The mice were manually guided to the platform if they failed to discover the platform. During the consolidation and retrial examination, the platform was invisible in a similar zone to the distant cue in the space. The probe examination was performed after the training duration to evaluate spatial memory. During the probe exam, each mouse was put in the third quadrant. The delay to the former platform position and the number of platform crossings are recorded for comparison. The investigator was blind to the treatment procedure.

The step down passive avoidance test (PA) was carried out as described previously (Xu et al., 2015). Briefly, each test was executed in a square chamber with a wooden platform on one part of the chamber’s grid floor. The floor can receive an electrical shock from an isolated pulse stimulator. Prior to training, the mice were allowed to adapt to the apparatus. During the adaptation procedure, each mouse was put on the platform, respectively. Once their feet were exposed to the grid floor, the mice suffered from a foot shock (20 s intertribal interval). The adaptation procedure was repeated by the training examination after 1 h. When they stayed on the platform for more than 60 s, the mice were considered to have taken the task in. Retention tests were performed 1 and 3 h after the training. During the retention duration, electrical shocks from the grid were taken away. Mice were put on a wooden platform solely, and the time of the first attempt to jump off the platform was documented as step-down latency with an upper limit of 300 s (Nasehi et al., 2012).

### Western Blot

Mice were decapitated after behavioral examinations. Related brain anatomical zones, specifically the prefrontal cortex and the hippocampus, were dissected. They were homogenized in lysis buffer and centrifuged at 12,000 rpm for 20 min at 4°C for protein measurement. The extracted total proteins (40 μg) were separated on 6, 10, or 15% SDS polyacrylamide gels using a vertical electrophoresis system (Bio-Rad, United States) and then transferred onto a nitrocellulose filter (NC) membrane using an electroblotting device (Bio-Rad, United States). The membranes were blocked for 1.5 h at room temperature with 2.5% BSA in TBST and then incubated with appropriate antibodies overnight at 4°C. The blots were incubated with peroxidase-conjugated secondary antibodies for 60 min at room temperature and visualized by enhanced chemiluminescence using a multifunctional chemiluminescence imaging system. The results of the Western blot analysis are representative of at least three experiments.

### Statistical Analysis

All data were expressed as means ± SEM. Unless specified otherwise, data were analyzed using one-way ANOVA and then subjected to *post hoc* Tukey’s test for multiple comparisons. The data from the acquisition trials in the MWM test were analyzed by two-way ANOVA. For the comparison between the two groups, data were analyzed using Student’s *t*-test. A *P* value of less than 0.05 was considered to be significant.

## Results

### Bay 60-7550 Reversed Aβ1–42-Induced Memory Impairment in the MWM Trial

**FIGURE 1:**
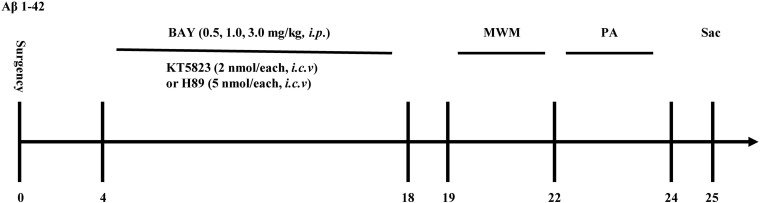
Experimental timeline for drug treatments. One and a half months after microinjection with Aβ1–42 into the cerebroventricle, mice were administrated with Bay 60-7550 for 14 days. H89 and KT5823 were pretreated 30 min before Bay 60-7550 administration every day. All the behavioral tests were performed 24 h after last drug treatment. Behavioral tests were carried out on days 19–24 and then the mice were sacrificed and biochemical assays were performed. MWM, Morris water maze; PA, passive avoidance test; Sac, sacrifice. *n* = 10 for each experiment.

**FIGURE 2:**
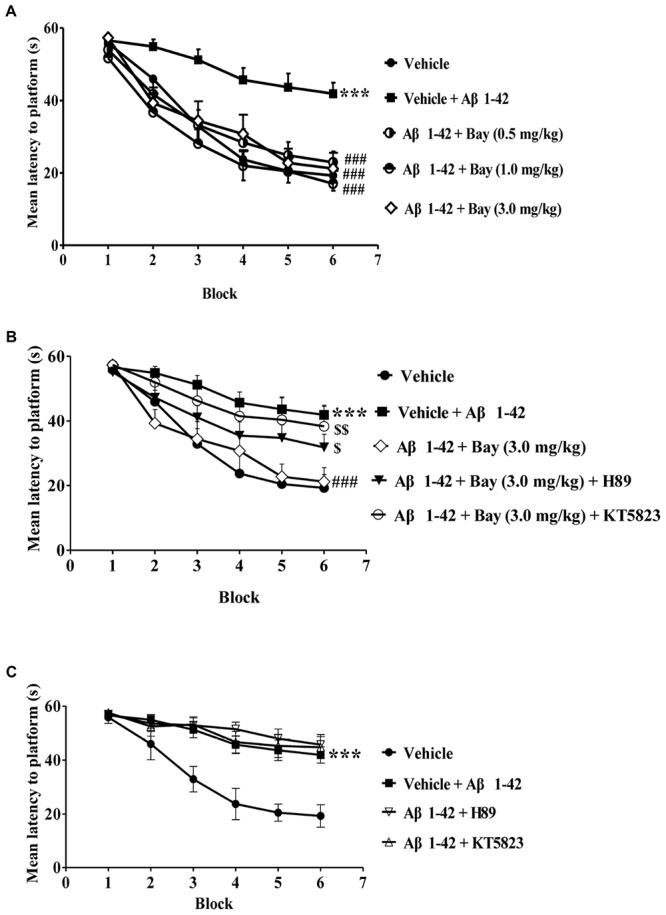
Learning curve in the water maze task after treatment with BAY 60-7550. **(A–C)** During the acquisition trials of the water maze task, the learning curve was tested 24 h after last treatment with Bay 60-7550. Values shown are means ± SEM, *n* = 10; ^∗∗∗^*p* < 0.001 vs. vehicle-treated sham group. ^###^*p* < 0.001 vs. vehicle-treated Aβ group. ^$^*p* < 0.05 and ^$$^*p* < 0.01 vs. Bay 60-7550 (3.0 mg/kg)-treated Aβ1–42 group.

The experimental procedure is shown in Figure 1. During the training section of six blocks in MWM, mice that received Aβ1–42 showed an increase in the average latency to the platform in block 6 as compared to the vehicle-treated control group (*F*_(__3__,__36__)_ = 22.93, *p* < 0.001, Figure 2A). Bay 60-7550 at doses of 0.5, 1.0, and 3.0 mg/kg by using intraperitoneal administration (i.p.) for 14 days decreased the average latency to the platform meaningfully in block 6 in Aβ1–42-treated mice (*F*_(__3__,__36__)_ = 27.61, Figure 2A). However, these results of Bay 60-7550 on memory acquisition were suppressed by PKG inhibitor KT5823 and PKA inhibitor H89, both of which were managed 30 min prior to Bay 60-7550 (Figure 2B). Furthermore, PKG inhibitor KT5823 or PKA inhibitor H89 that used alone had no effect on acquisition ability in block 6 in Aβ1–42-treated mice (Figure 2C).

**FIGURE 3:**
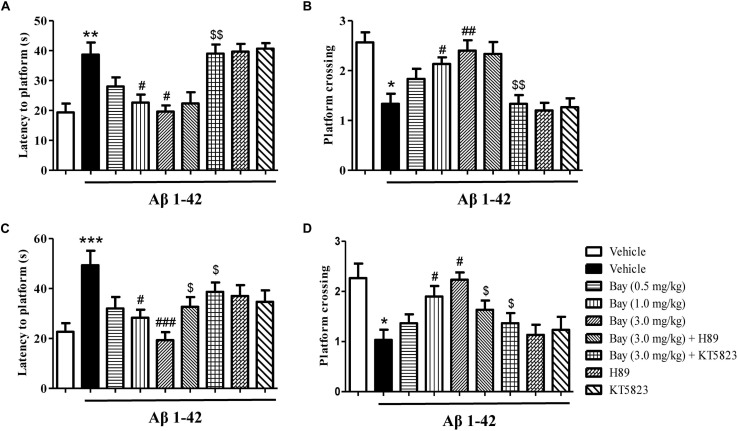
The effects of Bay 60-7550 on Aβ1–42-induced memory impairment in the Morris water maze (MWM) test in mice. The probe trial was conducted 1 h after the training; the latency to reach the platform **(A)** and the number of platform crossings **(B)** were determined. The probe trial was conducted 24 h after the training; the latency to reach the platform **(C)** and the number of platform crossings **(D)** were determined. Values shown are means ± SEM, *n* = 10; ^∗^*p* < 0.05, ^∗∗^*p* < 0.01, and ^∗∗∗^*p* < 0.001 vs. vehicle-treated sham group. ^#^*p* < 0.05, ^##^*p* < 0.01, and ^###^*p* < 0.001 vs. vehicle-treated Aβ group. ^$^*p* < 0.05 and ^$$^*p* < 0.01 vs. Bay 60-7550 (3.0 mg/kg)-treated Aβ1–42 group.

The probe examination was performed 1 h after training to evaluate short-term spatial memory (Figures 3A,B). The results indicated that the application of Aβ1–42, 2 months before the MWM examination, raised the latency to the former platform position and reduced the number of platform crossings as compared to the vehicle-treated controls. These effects were reversed by treatment of Bay 60-7550 at 1 or 3.0 mg/kg (Figures 3A,B). Long-term spatial memory was also identified in 24 h probe trials (Figures 3C,D). The Aβ1–42-treated mice’s memory performance was poor, illustrated by increased latency to the decreased platform crossings (Figure 3D) and previous platform location (Figure 3C). Bay 60-7550 at doses of 1.0 or 3.0 mg/kg ameliorated the effects of Aβ1–42 on latency to platform (Figure 3C) and platform crossings (Figure 3D). The ameliorating results of high-dose Bay 60-7550 (3.0 mg/kg) both in long-term and short memories were blocked by pretreatment with KT5823 (Figure 3). H89 prevented high-dose Bay 60-7550 (3.0 mg/kg)-induced long-term memory retention (24 h after the training session) (Figures 3C,D), but not short-term memory retrieval (1 h after the training session) in the water maze test (*p* > 0.05, Figures 3A,B), while neither H89 nor KT5823 used alone impacted the learning and memory abilities in Aβ1–42-treated mice (Figure 3).

### Bay 60-7550 Reversed Aβ1–42-Induced Aversive Memory Impairment in the PA Test

**FIGURE 4:**
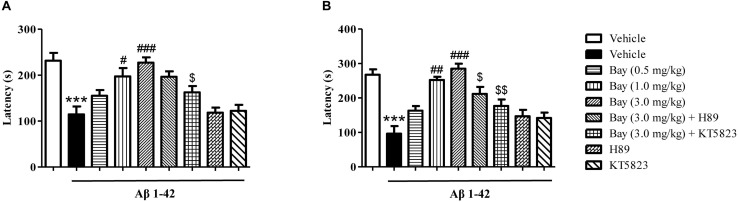
The effects of Bay 60-7550 on 1 h **(A)** and 3 h **(B)** memory retention in the step-down passive avoidance test in Aβ1–42-treated mice. Aβ1–42-induced decreases in 1 h and 3 h retention were reversed by chronic treatment with rolipram for 14 days. Values shown are means ± SEM, *n* = 10; ^∗∗∗^*p* < 0.001 vs. vehicle-treated control group. ^#^p < 0.05, ^##^p < 0.01, and ^###^p < 0.001 vs. vehicle-treated Aβ1–42 group. ^[*dollar*]^*p* < 0.05, ^[*dollar*][*dollar*]^*p* < 0.01 vs. Bay 60-7550 (3.0 mg/kg)-treated Aβ1–42 group.

Considering that the spatial memory deficits are always accompanied by emotional disorders such as depression and anxiety at the late stage of AD patients, the present study examined whether inhibition of PDE2 by Bay 60-7550 could ameliorate emotional memory disorder by the PA test. As shown in Figures 4A,B, animals exposed to Aβ1–42 showed a significant reduction in the step-down latency both 1 and 3 h after the training session (Figures 4A,B). Treatment of Bay 60-7550 (0.5, 1.0 and 3.0 mg/kg, i.p.) for 14 days enhanced memory retention (Figures 4A,B). PKG inhibitor KT5823 significantly prevented the effects of Bay 60-7550 on step-down latency both 1 and 3 h after the training session (Figures 4A,B). PKA inhibitor H89 significantly prevented the effects of Bay 60-7550 on step-down latency 3 h after the training session (Figure 4B), but not step-down latency 1 h after the training session (Figure 4A). Neither H89 nor KT5823 used alone impacted the memory ability in Aβ1–42-treated mice (Figures 4A,B).

### Bay 60-7550 Reduced the Ratio of Adrenal Gland to Body Weight (AG/B), but Did Not Change the Serum CORT Levels

**FIGURE 5:**
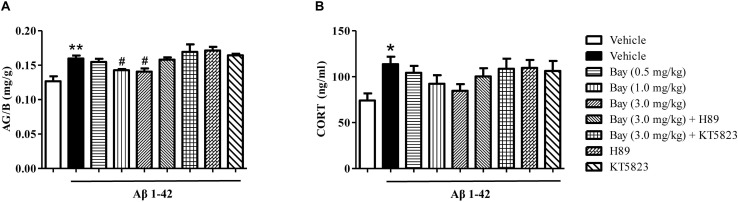
The effects of Bay 60-7550 on the ratio of the adrenal gland weight to body weight (AG/B) (mg/g) **(A)** and serum corticosterone level **(B)** in Aβ1–42-treated mice. Values shown are means ± SEM, *n* = 10; ^∗^*p* < 0.05, ^∗∗^*p* < 0.01 vs. vehicle-treated control group. ^#^*p* < 0.05 vs. vehicle-treated Aβ group.

The ratio of AG/B relative to vehicle-treated controls was significantly increased by Aβ1–42 (Figure 5A). However, Bay 60-7550 (1.0 and 3.0 mg/kg) significantly prevented Aβ1–42-induced increase in AG/B ratio as compared to the Aβ1–42 treated control groups (Figure 5A). Surprisingly, animals that were exposed to Aβ1–42 for 2 months only induced a tendency toward an increase in CORT level, while Bay 60-7550 also reduced this CORT level when compared to vehicle-treated Aβ1–42 treated control groups, although this reduction did not show significance (Figure 5B).

### Bay 60-7550 Reversed Aβ1–42-Induced Increases in GR Expression and CRF Receptor in the Hippocampus and Prefrontal Cortex

**FIGURE 6:**
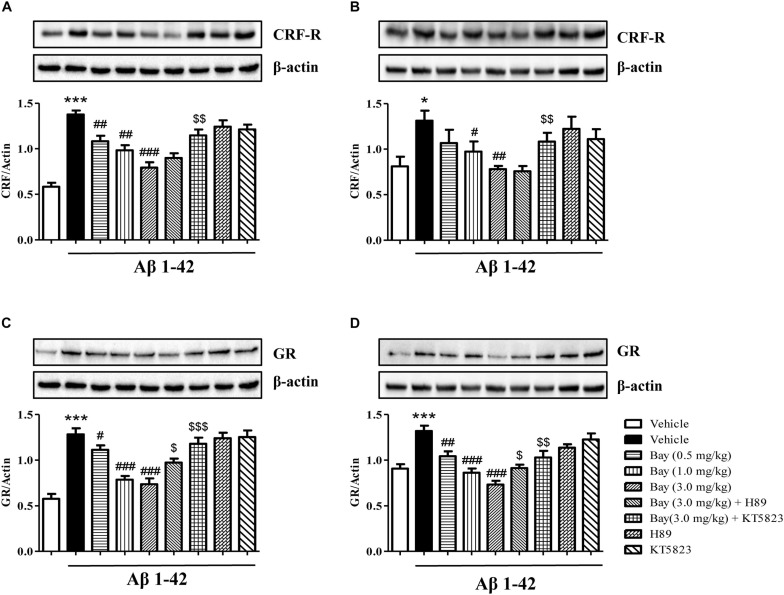
The effects of Bay 60-7550 on Aβ1–42-induced changes in CRF receptor and GR expression in the hippocampus **(A,C)** and in the cortex **(B,D)** of mice. Values shown are means ± SEM, *n* = 10; ^∗^*p* < 0.05 and ^∗∗∗^*p* < 0.001 vs. vehicle-treated control group. ^#^*p* < 0.05, ^##^*p* < 0.01, and ^###^*p* < 0.001 vs. vehicle-treated Aβ group. ^$^*p* < 0.05, ^$$^*p* < 0.01, and ^$$$^*p* < 0.001 vs. Bay (3.0 mg/kg)-treated Aβ group.

To determine whether inhibition of PDE2 could protect neurons against Aβ1–42-induced HPA dysfunction, the CRF receptor and GR expression were determined in the hippocampus and prefrontal cortex after treatment with Bay 60-7550. CRF receptor expression in Aβ1–42 treated mice showed a significant increase as compared to the respective control groups in the hippocampus and prefrontal cortex (Figures 6A,B). This increased CRF receptor levels both in the hippocampal (Figure 6A) and prefrontal cortex (Figure 6B) were significantly prevented by treatment with Bay 60-7550 for 14 days. However, these effects were blocked by pretreatment with PKG inhibitor KT5823 (Figures 6A,B), but not H89.

The GR levels were also increased in mice treated with Aβ1–42 both in the hippocampus and prefrontal cortex (Figures 6C,D). Chronic treatment with Bay 60-7550 at three different doses significantly blocked the Aβ1–42-induced GR increases in the hippocampus (Figure 6C) and prefrontal cortex (Figure 6D). However, both KT5823 and H89 blocked these Bay 60-7550’s effects on GR expression (Figures 6C,D), whereas neither H89 nor KT5823 used alone showed any effect on GR expression and CRF receptor.

### Bay 60-7550 Reversed Aβ1–42-Induced Decreases in BDNF and pCREB/CREB Expression in the Prefrontal Cortex and Hippocampus

**FIGURE 7:**
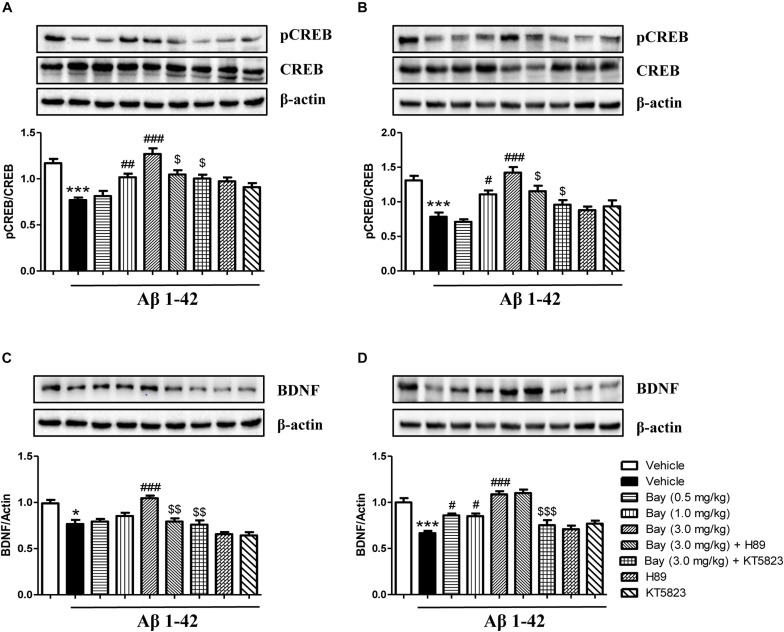
The effects of Bay 60-7550 on Aβ-induced changes in the ratio of pCREB to CREB and BDNF expression in the hippocampus **(A,C)** and the cortex **(B,D)** of mice. Values shown are means ± SEM, *n* = 10; ^∗^*p* < 0.05 and ^∗∗∗^*p* < 0.001 vs. vehicle-treated control group. ^#^*p* < 0.05, ^##^*p* < 0.01, and ^###^*p* < 0.001 vs. vehicle-treated Aβ group. ^$^*p* < 0.05, ^$$^*p* < 0.01, and ^$$$^*p* < 0.001 vs. Bay (3.0 mg/kg)-treated Aβ group.

Aβ1–42 induced brain damage and even neuronal death that are related to decreased CREB phosphorylation. In addition, subsequent neurotrophic factor expression such as decreased BDNF expression can also be affected. The reduction of pCREB/CREB ratio was observed in both the hippocampus and prefrontal cortex of mice after 2 months of Aβ1–42 exposure (Figures 7A,B). This reduction was ameliorated by treatment of Bay 60-7550 for 14 days (Figures 7A,B). However, both KT5823 and H89 blocked these Bay 60-7550’s effects on pCREB/CREB ratio in these two brain regions (Figures 7A,B).

The subsequent study suggested a significant reduction in BDNF expression of the hippocampus (*p* < 0.05, Figure 7C) and prefrontal cortex (*p* < 0.001, Figure 7D). This reduction was ameliorated by treatment of Bay 60-7550 for 14 days in both the hippocampus (*p* < 0.001, Figure 7C) and prefrontal cortex (*p* < 0.05, Figure 7D). PKG inhibitor KT5823 blocked Bay 60-7550’s effects on BDNF expression in these two brain regions (*p* < 0.01, Figure 7C; *p* < 0.001, Figure 7D). However, PKA inhibitor H89 significantly prevented the effects of Bay 60-7550 on BDNF expression in the hippocampus (*p* < 0.01, Figure 7C), but not in the prefrontal cortex (*p* > 0.05, Figure 7D).

## Discussion

In the present study, intracerebroventricular administration of Aβ1–42 caused overall impairment of memory and learning performance, accompanied by HPA axis dysfunction, decreased transcription factor (pCREB) and neurotrophic factor (BDNF) expression, at 2 months after treatment. Selective PDE2 inhibitor Bay 60-7550 was able to enhance Aβ1–42-induced cognitive and memory impairment through regulating the HPA axis, causing rises in BDNF levels and pCREB. By using the PKA inhibitor H89 or the PKG inhibitor KT5823, these effects, nevertheless, were blocked. These discoveries indicated that Bay 60-7550 improved memory and cognition through Aβ-induced HPA axis dysregulation, which caused the downstream cAMP/cGMP-dependent neuroprotective signaling pathway.

Aβ deposition in brain zones such as the prefrontal cortex and hippocampus is considered an early or late affair in the procedure of AD, which is involved in the worsening in memory processes and learning (Wang et al., 2016). Our previous studies suggested that the PDE4 inhibitor rolipram reversed Aβ1–42-induced memory impairment, as evidenced by increased crossing numbers and shorter latency to reach the target zone where the platform was removed in the test session (Xu et al., 2018). However, PDE4 inhibitors may cause severe nausea and vomiting, particularly at the high doses necessary for therapeutic benefit (Robichaud et al., 2002; Azzouni and Samra, 2011), while PDE2 presence was plentiful in the limbic nervous system (cortex and hippocampus) and the adrenal cortex (Van Staveren et al., 2003), which are areas predominantly relevant to HPA axis regulation and cognitive processes, while it does not cause nausea and vomiting. Moreover, some studies suggest that PDE2 inhibition reverses chronic stress- or Aβ-induced memory deficits (van Donkelaar et al., 2008; Reneerkens et al., 2013), which are consistent with our previous studies suggesting that PDE2 inhibition could reverse memory impairment and chronic stress-induced learning via the HPA axis’ regulation and the downstream signaling (Xu et al., 2015, 2018). The present study expanded upon our previous studies and demonstrated that Bay 60-7550 at doses of 0.5, 1.0, and 3.0 mg/kg daily for 14 days was able to protect animals against Aβ1–42-induced spatial memory deficits, as mice treated with Bay 60-7550 learned at a faster rate in both the training (acquisition of memory) and test sessions (1 and 24 h after the training session), i.e., ameliorated short- and long-term memory consolidation and retrieval in the water maze test. These outcomes were supported by the subsequent step-down passive avoidance test, which indicated that short-term (1 h) and long-term (3 h) aversive memory retention was ameliorated by treatment with Bay 60-7550. Pretreatment of animals with either the PKA inhibitor H89 or the PKG inhibitor KT5823, nevertheless, inhibited Bay 60-7550 induced memory acquisition as demonstrated in the learning curve, which are partially contradictory with previous studies that suggested that only the PKG inhibitor KT5823 prevented Bay 60-7550 induced memory-enhancing effect (Wang et al., 2017). The possible reason is the big fluctuation of behavioral test between animals. Moreover, H89 prevented Bay 60-7550-induced long-term memory retention (24 h after the training session), but not short-term memory retrieval (1 h after the training session) in the water maze test. However, KT5823 reversed both the short-term and long-term memory retention in the water maze test. These are partially contradictory with the previous findings in which cGMP enhancement is critical for short-term memory retention, while cAMP enhancement is mainly responsible for long-term memory retention (Bollen et al., 2014). Indeed, although PDE2 hydrolyzes both cAMP and cGMP, the enzyme activity is increased and cGMP synthesis is elevated (Wu et al., 2004; Lugnier, 2006). For example, the affinity of PDE2 toward cGMP is more than 30 times higher than that of cAMP. Thus, cAMP signaling did not affect short-term memory in the present study, and it could be due to the functional activation of cAMP signaling requiring higher doses of PDE2 inhibitor or longer treatment time. Recent studies conducted exploratory experiments and updated the concept of the memory process, which suggests that both cAMP-PKA and cGMP-PKG signaling pathways play crucial roles in the short- and long-term memory formation and consolidation (Wang et al., 2017). In addition, the indirect upregulation of silent information regulator 1 (SIRT1) that is essential for cognitive enhancement and synaptic plasticity after treatment with Bay 60-7550 may stimulate transcription factor such as CREB, which may act as the molecular switch from short- to long-term memory (Zhao et al., 2019).

Recent evidence showed that microinjection of Aβ fragment (oAβ) into the cerebroventricle induces a wide pattern of pathological changes in the brain including long-lasting overactivation of the limbic–HPA axis that is parallel to the pathophysiology of human AD (Zussy et al., 2013; Pineau et al., 2016). Our present study suggested that intracerebroventricular administration of Aβ1–42 caused significant increases in the ratio of the adrenal gland to body weight (AG/B), serum CORT level, as well as CRF-R and GR expression in the hippocampus and prefrontal cortex. These Aβ1–42-induced abnormalities were reversed by treatment with Bay 60-7550 except for the CORT levels. Indeed, the HPA axis is highly involved in Aβ-initiated stress response and triggers the adrenal cortex to release glucocorticoids (CORT in rodents). These steroid hormones readily cross the blood–brain barrier and activate GR and CRF, resulting in negative feedback dysfunction in the HPA axis (Reul and de Kloet, 1985). Our former researches indicated that the dysregulation of the HPA axis reduced the levels of the second messenger cyclic nucleotides (cGMP and cAMP) (Xu et al., 2013, 2015), subsequently decreasing the phosphorylation of CREB and BDNF expression, which, in turn, worsens memory and learning disorders. The present research indicated that the inhibition of PDE2 raised cAMP/cGMP levels and irritated HPA axis negative feedback regulation, causing reductions in CORT releasing and CRF receptor and GR expression. Furthermore, the present study suggested that Bay 60-7550 did not impact the CORT level significantly, which may result from the large fluctuation of serum CORT values between animals. Further studies are ongoing to confirm whether Bay 60-7550 prevented CORT levels in the brain or serum when animals were exposed to Aβ1–42 at different time points.

In the animal model of AD, cognitive deficits are often accompanied by the formation of Aβ plaques, which deactivates the cAMP/cGMP pathway by decreasing the phosphorylation of CREB and BDNF expression (Xu et al., 2006). Moreover, increasing evidence indicates that cAMP/cGMP analogs counteract the Aβ-induced decrease in CREB phosphorylation. The present research suggested that Aβ-induced decreases in the ratio of pCREB/CREB and BDNF expression were rescued by the PDE2 inhibitor Bay 60-7550. These effects were reversed by KT5823 and, to a lesser extent, by H89. This further supports that the cAMP/cGMP-pCREB-BDNF pathway is involved in the protective effects of Bay 60-7550 on Aβ-induced memory deficits.

In conclusion, the present study indicated that microinjection of Aβ1–42 to the cerebroventricle caused learning and memory damages and dysregulation of the HPA axis. The PDE2 inhibitor Bay 60-7550 significantly ameliorated the learning and memory loss by regulation of the HPA axis and the downstream cAMP/cGMP-pCREB-BDNF signaling. Assessing this PDE2 inhibitor gave rise to opportunities to reveal the processes underlying Aβ relevant learning and memory deficits, which, in turn, may uncover strategies for the treatment of age-related disorders, such as AD.

## Data Availability Statement

The datasets generated for this study are available on request to the corresponding authors.

## Ethics Statement

The animal study was reviewed and approved by the Animal Care and Use Committee of Wenzhou Medical University.

## Author Contributions

LR, KD, and MT wrote the manuscript and did a part of the behavioral tests. CS, RY, and YT performed the biochemical and neurobiological experiments. HP and JL analyzed the data and revised the manuscript. MZ and JP designed and supervised the manuscript.

## Conflict of Interest

The authors declare that the research was conducted in the absence of any commercial or financial relationships that could be construed as a potential conflict of interest.
